# Personalized prevention for all: changing how we approach the future of prevention

**DOI:** 10.1093/haschl/qxag089

**Published:** 2026-04-16

**Authors:** Robb Rowley, Nephi A Walton

**Affiliations:** Division of General Internal Medicine (Rowley), Mayo Clinic, Rochester, MN 55905, United States; Department of Epidemiology (Walton), Wake Forest University School of Medicine, Winston-Salem, NC 27101, United States; Division of General Internal Medicine (Rowley), Mayo Clinic, Rochester, MN 55905, United States; Department of Epidemiology (Walton), Wake Forest University School of Medicine, Winston-Salem, NC 27101, United States

**Keywords:** genomics, health care costs, health savings accounts, integrated risk, personalized prevention

## Abstract

Advances in genomics, informatics, and artificial intelligence (AI) are reshaping preventive medicine by enabling risk-stratified, personalized approaches to disease prevention. This paper argues for a systemic transformation in US health care policy to support equitable access to personalized prevention. We critique the inefficiencies of current age-based screening paradigms and highlight the potential of integrating polygenic risk scores, multi-omic data, and AI-driven analytics to optimize preventive strategies. However, without concurrent innovation in financing models, these advances risk exacerbating existing health disparities. Drawing on lessons from the rare disease community, we propose policy solutions, including expanded, equitably funded health savings accounts, transparent pricing, and patient-centered decision-support tools, to democratize access to personalized prevention. We advocate for a healthcare ecosystem that aligns technological innovation with flexible, patient-directed funding mechanisms, enabling all persons, not just the well-resourced, to benefit from precision prevention. The Human Genome Project promised to unlock the complete genetic blueprint of humans to revolutionize medicine through personalized care, disease prevention, and new biotechnologies. Realizing this goal requires coordinated action across science, policy, and payment systems to ensure that personalized prevention becomes a standard, equitable component of modern health care.

Genomics and related innovations, including artificial intelligence (AI), are reshaping preventive medicine by making it increasingly possible to tailor further prevention to individual risk. These advances could improve outcomes, but without payment and policy reform they may disproportionately benefit patients with greater financial means and better access to advanced care. Current insurance models often exclude preventive strategies because coverage depends on lengthy evidence review and guideline adoption. Steps are therefore needed to prevent personalized prevention from worsening health disparities.

Here, we advocate for broader adoption of personalized prevention, outlining its rationale, enabling factors, and the need for more flexible approaches to financing preventive care. We then propose strategies to expand access so that more patients can benefit from advances in disease risk assessment and management.

## The rationale for personalized prevention

Current prevention is largely one-size-fits-all, overlooking individualized approaches enabled by genomics and informatics. Colon cancer screening is one example. The US Preventive Services Task Force (USPSTF) lowered the screening age from 50 to 45 years, a change projected to reduce incidence by 7117 annually.^1^ Yet, this expansion means that about 21.4 million adults age 45 to 49 will undergo screening many at relatively low short-term risk. This does not argue against age-based screening, which has a clear population benefit, but it illustrates the limits of relying on age alone when risk varies across individuals. Conversely, individuals with markedly increased risk, such as those with Lynch syndrome remain underdiagnosed despite evidence that earlier colonoscopies can reduce lifetime cancer risk by 60% to 70%.^2-4^ This mismatch highlights why prevention policy must better account for heterogeneity in risk.

More efficient prevention requires better individual risk assessment. Expanding health data and improved informatics are refining how risk is estimated and how preventive strategies are matched to patients most likely to benefit. While age has long served as a simple risk marker, genomics and related tools now allow more nuanced estimates. The WISDOM study, for example, risk-stratifies patients for personalized breast cancer screening and illustrates the value of tailored prevention.^5^

## Factors advancing personalized prevention

Digital health monitoring, growing health data, and improved analytics are accelerating personalized prevention by enabling more tailored interventions. These developments are evident in colon cancer prevention, where options now include endoscopy, stool DNA tests, and emerging multicancer detection technologies.^4^ More broadly, personalized prevention is being advanced by digitized health records, wearable devices, telemedicine, multi-omic data, imaging, biomarkers, and patient-generated health data.

Polygenic risk scores (PRS) may improve early risk stratification in some settings, but their limitations must be explicit. PRS performance varies across populations, particularly when models developed largely in people of European ancestry are applied to individuals of different genetic ancestry or other underrepresented ancestries.^6, 7^ For this reason, PRS should not be treated as deterministic or used in isolation. Rather they should be interpreted as one part of a broader risk framework that also includes clinical history, family history, biomarkers, and other contextual factors. Recent studies, including BARCODE1 and WISDOM, suggest that risk informed screening may have clinical utility, but the evidence is still evolving and requires continued validation across diverse populations.^8, 9^

Navigating these options will require patients and clinicians to determine the timing, type, and frequency of preventive interventions according to individual risk and preferences. Clinician training in quantitative risk estimation, including interpretation and limitations of PRS and other predictive models, will be essential as these tools move into clinical practice. Without such training, implementation could unintentionally reinforce inequities.

To move away from rigid, one-size-fits-all insurance criteria, the health care system must enable individuals and clinicians to choose among preventive strategies tailored to the patients’ risk and values and after an informed discussion. In colorectal cancer, for example, genomic and clinical data could guide who should be screened earlier, more intensively, or by different methods.

## Individualized demands of personalized prevention

A central challenge for personalized prevention is how to fund and sustain it. Insurers have long been reluctant to pay for preventive measures, which is why the Affordable Care Act (Section 2713), requires private insurers to cover USPSTF “A” or “B” rated services.^1^ Yet, there are often delays between scientific recognition and formal coverage. *BRCA* testing, for example, was recognized by the CDC in 2012 but not formally recommended by the USPSTF until 2019. As prevention becomes increasingly personalized, coverage may become even harder because many strategies will apply to smaller groups defined by combinations of genetic, clinical, and environmental risk factors.

The rare disease community offers valuable lessons. For decades, patients with rare diseases have faced barriers to care tailored to their needs because targeted interventions often do not fit conventional evidence standards.^10^ Personalized prevention may face a similar problem: many patients may benefit from preventive strategies that fall outside current guideline-based coverage. These parallels underscore how individualized prevention may challenge payment systems built around standardized recommendations.

## Leveraging information access and AI

Digital tools, including AI-enabled decision support, may help patients and clinicians interpret increasingly complex risk information, but their value depends on validation, transparency, and careful integration into clinical decision-making.^11-18^ Any AI-supported approach should be evaluated on clinical performance, fairness across populations, and its ability to improve shared decision-making rather than replace it.

Tension arises when shared decisions leads to preventive strategies outside insurance-approved guidelines. One response is to build informatic systems that catalog services, support transparent pricing, and present evidence relevant to each person's risk profile. Such tools could help patients and clinicians make informed choices even before insurers or guidelines adapt, although they will matter only if patients also have realistic means to pay for these services.

## Financing flexibility for personalized prevention

Promoting transparent pricing, simplifying option comparison, and offering greater flexibility to choose tailored strategies will help realize personalized prevention. Expanding access may also encourage development of tools and services that help patients and clinicians navigate growing numbers of risk models and preventive interventions.

Health savings accounts (HSAs) illustrate one possible mechanism for giving patients flexibility to pay for preventive services that do not fit neatly into current coverage rules, but HSAs are not sufficient on their own.^19^ Evidence from high-deductible plan designs suggests that when patients face greater cost sharing, they often reduce both high- and low-value care rather than selectively reducing low-value services.^20^ Related evidence suggests that higher cost sharing can delay needed outpatient care and worsen outcomes, underscoring that financing flexibility alone does not ensure clinically sound decision-making.^21^

HSAs could help fund personalized prevention, and enrollment has grown substantially.^22^ However, the existing literature shows that HSAs disproportionately benefit higher income households that can afford to contribute and capture the tax advantages. Any equitable adaptation of this model would therefore require direct support for lower-income patients. The level and structure of such support would require further policy deliberation. However, the challenge of the HSA model illuminates is not unique to HSAs. For example, Medicaid coverage for genetic testing remains highly variable across states, indications, and billing pathways, illustrating how payer fragmentation creates uneven access to personalized services.^23, 24^ Any financing approach for personalized prevention must therefore be paired with clinician guidance, patient education, and decision support tools if we will be able to effectively use their finances to improve patients’ health through prevention.

More flexible financing could still support informed patient choice and encourage development of more accessible preventive service models. The goal is not to shift prevention entirely to out-of-pocket purchasing, but to create practical pathways for patients to access evidence informed services that fall outside rigid coverage structures.

## Conclusion

Personalized prevention represents a shift from rigid, population-based third party-payer-fee structures toward strategies tailored to individual risks, preferences, and circumstances. Advances in genomics, informatics, and AI make such customization increasingly possible, but without parallel innovation in financing, access will remain limited to those who can pay out of pocket. Failing to address this gap risks turning personalized prevention into a driver of inequity rather than a means of improving prevention.

By adopting mechanisms that promote transparent pricing, facilitate comparison of options, and provide more flexible and equitable financing, health systems can better support shared decision-making and broader access to tailored prevention. The Human Genome Project promised a future in which understanding each person's biology would enable more precise and effective disease prevention. That promise will be realized only if science, payment policy, and innovation evolve together so that personalized prevention becomes a broadly available standard rather than a privilege.

## Supplementary Material

qxag089_Supplementary_Data

## References

[1] U.S. Preventive Services Task Force . Procedure Manual Appendix I. Congressional Mandate Establishing the U.S. Preventive Services Task Force. Accessed December 9, 2025. https://www.uspreventiveservicestaskforce.org/uspstf/about-uspstf/methods-and-processes/procedure-manual/procedure-manual-appendix-i#:∼:text=the Task Force.-,Sec.,any cost sharing requirements for%E2%80%94.

[2] Lindor NM, Petersen GM, Hadley DW, et al Recommendations for the care of individuals with an inherited predisposition to lynch syndrome: a systematic review. JAMA. 2006;296(12):1507–1517. 10.1001/jama.296.12.1507 17003399

[3] Knudsen AB, Rutter CM, Peterse EFP, et al Colorectal cancer screening: an updated modeling study for the US preventive services task force. JAMA. 2021;325(19):1998–2011. 10.1001/jama.2021.5746 34003219 PMC8409520

[4] Ladabaum U, Dominitz JA, Kahi C, Schoen RE. Strategies for colorectal cancer screening. Gastroenterology. 2020;158(2):418–432. 10.1053/j.gastro.2019.06.043 31394083

[5] Eklund M, Broglio K, Yau C, Connor JT, Stover Fiscalini A, Esserman LJ. The WISDOM personalized breast cancer screening trial: simulation study to assess potential bias and analytic approaches. JNCI Cancer Spectr. 2018;2(4):pky067. 10.1093/jncics/pky067 31360882 PMC6649825

[6] Kachuri L, Chatterjee N, Hirbo J, et al Principles and methods for transferring polygenic risk scores across global populations. Nat Rev Genet. 2024;25(1):8–25. 10.1038/s41576-023-00637-2 37620596 PMC10961971

[7] Chin MH, Afsar-Manesh N, Bierman AS, et al Guiding principles to address the impact of algorithm bias on racial and ethnic disparities in health and health care. JAMA Netw Open. 2023;6(12):e2345050. 10.1001/jamanetworkopen.2023.45050 38100101 PMC11181958

[8] McHugh JK, Bancroft EK, Saunders E, et al Assessment of a polygenic risk score in screening for prostate cancer. N Engl J Med. 2025;392(14):1406–1417. 10.1056/NEJMoa2407934 40214032 PMC7617604

[9] Esserman LJ, Fiscalini AS, Naeim A, et al Risk-based vs annual breast cancer screening: the WISDOM Randomized Clinical Trial. JAMA. 2026;335(9):763–774. 10.1001/jama.2025.24784 41385349 PMC12701531

[10] The National Organization for Rare Disorders . Barriers to Rare Disease Diagnosis, Care and Treatment in the US: A 30-Year Comparative Analysis. 2020. Accessed December 9, 2025. https://rarediseases.org/wp-content/uploads/2020/11/NRD-2088-Barriers-30-Yr-Survey-Report_FNL-2.pdf.

[11] Iqbal U, Celi LA, Li YJ. How can artificial intelligence make medicine more preemptive? J Med Internet Res. 2020;22(8):e17211. 10.2196/17211 32780024 PMC7448175

[12] Warraich HJ, Tazbaz T, Califf RM. FDA perspective on the regulation of artificial intelligence in health care and biomedicine. JAMA. 2025;333(3):241–247. 10.1001/jama.2024.21451 39405330

[13] Angus DC, Khera R, Lieu T, et al AI, health, and health care today and tomorrow: the JAMA summit report on artificial intelligence. JAMA. 2025;334(4):1650–1664. 10.1001/jama.2025.18490 41082366

[14] Clark P, Kim J, Aphinyanaphongs Y. Marketing and US Food and Drug Administration clearance of artificial intelligence and machine learning enabled software in and as medical devices: a systematic review. JAMA Netw Open. 2023;6(7):e2321792. 10.1001/jamanetworkopen.2023.21792 37405771 PMC10323702

[15] El Sherbini A, Rosenson RS, Al Rifai M, et al Artificial intelligence in preventive cardiology. Prog Cardiovasc Dis. 2024;84:76–89. 10.1016/j.pcad.2024.03.002 38460897

[16] Parsa S, Somani S, Dudum R, Jain SS, Rodriguez F. Artificial intelligence in cardiovascular disease prevention: is it ready for prime time? Curr Atheroscler Rep. 2024;26:263–272. 10.1007/s11883-024-01210-w 38780665 PMC11457745

[17] Muse ED, Topol EJ. Transforming the cardiometabolic disease landscape: multimodal AI-powered approaches in prevention and management. Cell Metab. 2024;36:670–683. 10.1016/j.cmet.2024.02.002 38428435 PMC10990799

[18] Rakers MM, van Buchem MM, Kucenko S, et al Availability of evidence for predictive machine learning algorithms in primary care: a systematic review. JAMA Netw Open. 2024;7:e2432990. 10.1001/jamanetworkopen.2024.32990 39264624 PMC11393722

[19] U.S. Department of Health & Human Services . Understanding Health Savings Account-eligible plans. 2025. Accessed December 9, 2025. https://www.healthcare.gov/high-deductible-health-plan/.

[20] Brot-Goldberg ZC, Chandra A, Handel BR, Kolstad JT. What does a deductible do? The impact of cost-sharing on health care prices, quantities, and spending dynamics. Q J Econ. 2017;132(3):1261–1318. 10.1093/qje/qjx013

[21] Wharam JF, Zhang F, Eggleston EM, Lu CY, Soumerai S, Ross-Degnan D. Diabetes outpatient care and acute complications before and after high-deductible insurance enrollment: a natural experiment for translation in diabetes (NEXT-D) study. JAMA Intern Med. 2017;177(3):358–368. 10.1001/jamainternmed.2016.8411 28097328 PMC5538022

[22] American Bankers Association . Survey: More Americans using health savings accounts. Accessed December 9, 2025. https://bankingjournal.aba.com/2023/07/survey-more-americans-using-health-savings-accounts/.

[23] Huston-Paterson HH, Mora V, Karlan BY, Teshome M. Medicaid coverage of NCCN- and ASCO/SSO-guideline-concordant BRCA germline testing for patients with breast cancer: opportunity to embrace rapid advancements in precision medicine. Ann Surg Oncol. 2025;32(2):646–649. 10.1245/s10434-024-16306-5 39580377

[24] Streff H, Uhles CL, Fisher H, et al Access to clinically indicated genetic tests for pediatric patients with medicaid: evidence from outpatient genetics clinics in Texas. Genet Med. 2023;25(3):100350. 10.1016/j.gim.2022.11.018 36547467

